# Health inequality among different economies during early phase of COVID-19 pandemic

**DOI:** 10.1186/s42506-021-00067-0

**Published:** 2021-02-17

**Authors:** Myo Nyein Aung, Yuka Koyanagi, Motoyuki Yuasa

**Affiliations:** 1 Juntendo Advanced Research Institute for Health Science, Juntendo University, 2-1-1, Hongo, Bunkyo-ku, Tokyo, 113-8421 Japan; 2 Global Health Service, Faculty of International Liberal Arts, Juntendo University, 2-1-1, Hongo, Bunkyo-ku, Tokyo, 113-8421 Japan; 3 Tokyo Ariake University of Health Science, Tokyo, Japan; 4 Department of Public Health, Faculty of Medicine, Juntendo University, 2-1-1, Hongo, Bunkyo-ku, Tokyo, 113-8421 Japan

**Keywords:** SDGs, COVID-19, HCoV-19, SARS-CoV-2, Health inequality, Mitigation

## Abstract

**Background:**

The new coronavirus outbreak originated in Wuhan, China, started in January 2020 is escalating as a pandemic across the globe in March 2020. It causes unprecedented morbidity and shocked health systems and the supply chains in new epicenters such as Italy, Spain, and the USA, claiming thousands of lives. Meanwhile, the pandemic is reaching swiftly and silently to low-income countries where international media cover less. How likely health outcomes among the countries with different economies may differ during the pandemic has not been reported yet. Methodologically, we conducted an analysis of COVID-19 deaths comparing case fatality rate (CFR) among countries with different income categories, applying COVID-19 global data from the European Centre for Disease Control including 199 countries’ data as of 31 March 2020, in the early phase of the pandemic. We categorized countries into high-income countries (HIC), upper-middle-income countries (UMIC), lower-middle-income countries (LMIC), and low-income countries (LIC) according to World Bank classification by income as of 2020.

**Finding:**

Statistically, countries in different income groups are significantly different in terms of new cases identified in the last 2 weeks and the case fatality rate (MANOVA, *P* value < 0.001). New tests and detected case numbers shot up in HICs where CFR shot up in LMICs and LICs. The results of this analysis pointed out an important gap among countries with different economic status during the ongoing pandemic.

**Conclusion:**

In the HIC, contact tracing, testing capacity, and outbreak response, as well as clinical services, are strong. In the LICs, there is a low capacity of outbreak response which is reflected by the significantly lower number of diagnostic tests. Consequently, the reported number of COVID-19 cases in LICs may not reflect the actual burden of the pandemic. Without effective prevention, the pandemic can readily break into the weak health system and over-burden the hospitals and clinical services in poor countries.

This finding is showing health inequality between the rich and the poor being amplified by the COVID-19 pandemic. Addressing such a gap through the local governance and integrated global responses will not only prevent unprecedented deaths, but also preserve the momentum towards Sustainable Development Goals (SDGs).

## Background

The new coronavirus outbreak originated in Wuhan, China, started in January 2020 is escalating as a pandemic across the globe in March 2020 [1, 2]. It causes unprecedented morbidity and shocked health systems and the supply chains in new epicenters such as Italy, Spain, and the USA, claiming thousands of lives. Meanwhile, the pandemic is reaching swiftly and silently to low-income countries where international media cover less. How likely health outcomes among the countries with different economies may differ during the pandemic has not been reported yet.

Health inequality can happen in terms of (1) access to diagnostic and treatment, (2) health outcomes such as death and recovery, (3) social determinants of health such as education, economic stability, neighborhood, social and community context, and health service delivery [3]. Pandemics usually undermined the goals of humanity achieved before the pandemic. While this paper is being written, the COVID-19 pandemic first wave had spread to every continent of the world within 3 months.

The objective of this study was to compare the number of COVID-19 cases and the death among diagnosed cases across the countries in the world, from the beginning of the pandemic until April 1, 2020. Ultimately, we aimed to highlight the inequality in access to diagnostic tests and COVID-19 outcomes in the spectrum of economies.

## Methods

We conducted an analysis of COVID-19 deaths comparing case fatality rate (CFR) among countries with different income categories, applying COVID-19 global data from the European Centre for Disease Control including 199 countries as of 31 March 2020 [4]. We categorized countries into high-income countries (HIC), upper-middle-income countries (UMIC), lower-middle-income countries (LMIC), and low-income countries (LIC) according to World Bank classification by income as of 2020 [5]. Stata version 16 (StataCorp, Special Edition College Station, TX, 77845, USA) was applied to analyze the data [6]. Ethical approval is not required when using open-source secondary data without identity.

## Results

**Fig. 1:**
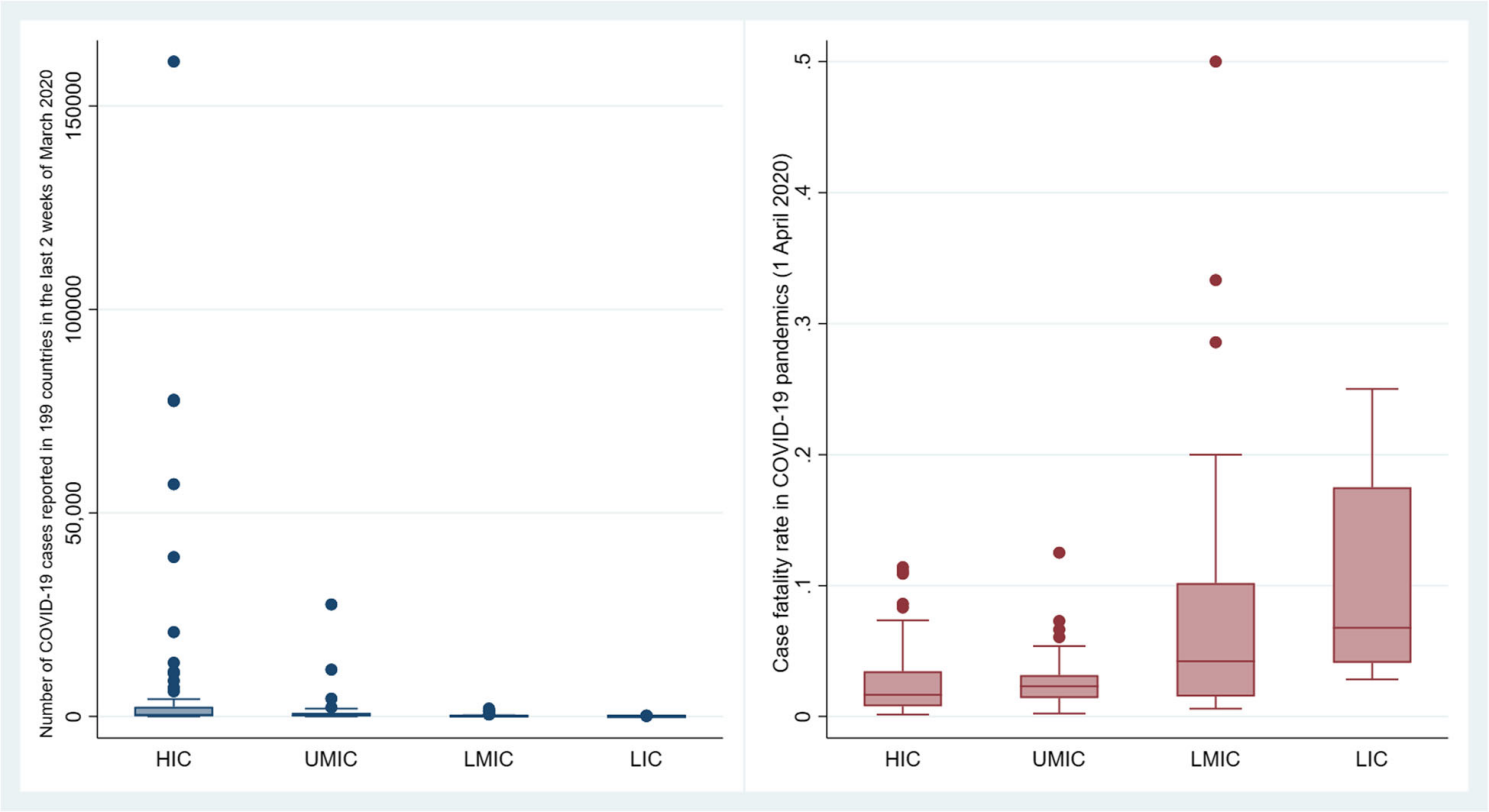
New cases of COVID-19 (left) and death rate (right) observed globally in countries with different income status as of 1 April 2020. HIC, high-income countries; UMIC upper-middle-income countries; LMIC, lower-middle-income countries; LIC low-income countries

Descriptive analysis showed obvious differences in new cases identified in the previous 2 weeks overshooting in HIC whereas CFR overshooting in LMIC and LIC (Fig. 1).

Statistically, countries in different income groups are significantly different in terms of new cases identified in the last 2 weeks and death rate (MANOVA, *P* value < 0.001). This finding is showing health inequality between the rich and the poor being amplified by the COVID-19 pandemic.

## Discussion

The World Health Organization encouraged testing and identification of new cases [7, 8]. Currently, a relatively low number of new cases identified in LMIC and LIC may reflect either the start of the epidemic curve or low testing capacity [2]. Moreover, CFR indicated how vulnerable are the health systems to a pandemic.

In the HIC, contact tracing, testing capacity, and outbreak response, as well as clinical services, are strong [9]. In the LICs, there is a low capacity of outbreak response which is reflected by the significantly lower number of diagnostic tests [10]. Consequently, the reported number in LICs may not reflect the actual burden of the pandemic. Without effective prevention, the pandemic will readily break the weak health system and over-burden the hospitals and clinical services [11].

Mitigation and suppression measures interact with businesses, culture, and health literacy in each population whereas testing capacity and health service resilience really depend on economic status. These inevitable temporary measures also will hinder many of LIMCs and LICs in the journey of sustainable development goals (SDGs) [5, 12]. The pandemic can impair the social determinants of the health and worsen existing inequalities [13]. However, it is still overlooked in many places [12].

The results of this analysis pointed out an important gap among countries with different economic status during the ongoing pandemic. Addressing such a gap through the local governance and integrated global responses will not only prevent unprecedented deaths, but also preserve the momentum towards SDGs. Furthermore, our finding is a signal that policy and implementation for strengthening the health system are to be timely in order to prevent overloading health service capacity. A collective action of prevention is more important than ever before to minimize the inequality getting more serious [14]. Thus, empowerment of the communities is critically important [3].

This paper has limitations. It was prepared very early in the first wave of the COVID-19 pandemic. The data analyzed in this study is confined to the first wave of the COVID-19 pandemic. Although our finding visually pointed out the inequalities of testing capacity for COVID-19 and CFR, we did not conduct any statistical analysis associating these two. Future studies are required to prove it. World Health Organization declared the SARS-CoV-2 outbreak as a pandemic on March 12, 2020 [15]. We analyzed global data on April 1, 2020. As this analysis was conducted in the early phase of the COVID-19 pandemic, the findings may reflect a natural history of the COVID-19 pandemic and health inequalities evolving into a global outbreak.

## Conclusion

To turn down the pandemic curve is an urgent global target. Further, seriously challenged health services and civil participation will leave vulnerable populations in profound death, poverty, hunger, and chaos. Therefore, we call for international collaborative efforts to save humanity and leaving no one behind towards SDGs.

## Data Availability

We can share the data we have used to conduct this research upon request, and the data set used in the analysis will be delivered.

## Abbreviation

CFR: Case fatality rate
HIC: High-income countries
LMIC: Lower-middle-income countries
LIC: Low-income countries
UMIC: Upper-middle-income countries
